# From Co‐Design to Quality Improvement: Using the Double Diamond Framework to Develop Primary Care Redesign Priorities for Hypertension Care

**DOI:** 10.1111/hex.70673

**Published:** 2026-04-19

**Authors:** Sonal J. Patil, Aleece Caron, Mary Joan Roach, Douglas Einstadter, Shelia Malone, Quisha Marbury, Morgan Whaley, Catherine Sullivan, Perry J. Kimmel, Marquita Rockamore, Christopher Mundorf, Kipum Lee, Shari D. Bolen

**Affiliations:** ^1^ Department of Family Medicine MetroHealth System and Case Western Reserve University, MetroHealth Community Health Centers Cleveland Ohio USA; ^2^ Center for Health Equity, Engagement, Education and Research MetroHealth System and Case Western Reserve University Cleveland Ohio USA; ^3^ Center for Health Care Research and Policy MetroHealth Population and Health Research Institute Cleveland Ohio USA; ^4^ Department of PM&R MetroHealth System and Case Western Reserve University School of Medicine Cleveland Ohio USA; ^5^ MetroHealth Centre for Rehabilitation Research, MetroHealth System Cleveland Ohio USA; ^6^ Department of Internal Medicine MetroHealth System and Case Western Reserve University Cleveland Ohio USA; ^7^ Better Health Partnership University Heights Ohio USA; ^8^ Cuyahoga Community College Cleveland Ohio USA; ^9^ UH Ventures University Hospitals Cleveland Ohio USA; ^10^ Department of Medicine Case Western Reserve University School of Medicine Cleveland Ohio USA; ^11^ Weatherhead School of Management Case Western Reserve University Cleveland Ohio USA

**Keywords:** clinic–community linkages, double diamond, health disparities, hypertension, primary care redesign, quality improvement, solution‐oriented approaches

## Abstract

**Introduction:**

Despite evidence supporting clinic‐community linked hypertension interventions for socially disadvantaged populations, sustaining these interventions in real‐world primary care has been challenging. The objective of this work was to identify shared potential priorities for redesigning hypertension care using a structured Double Diamond co‐design framework across health systems and community‐based organizations (CBOs) serving census tracts with a high prevalence of hypertension.

**Methods:**

We used the Double‐Diamond design strategy to co‐create hypertension care redesign priorities with four diverse health systems and multiple CBOs from our target census tracts. We established two co‐design working groups: one for data and evaluation elements, and one for community engagement and health equity elements. Fourteen structured co‐design meetings followed the Double Diamond phases (discover, define, develop, deliver) to identify needs and refine priorities. We used thematic analysis and triangulated findings from meeting summaries, fieldwork observations, existing community health needs assessments and geospatial data assessments of the priority census tracts to identify shared priorities for quality improvement (QI) efforts across health systems.

**Results:**

Our target census tracts had greater socioeconomic disadvantages compared to surrounding tracts. Stakeholders identified actionable redesign priorities, including improving data usability and standardizing data across health systems; incorporating the lived experiences of patients; enhancing interoperability and the real‐time availability of CBO resources; and fostering cross‐system team‐based care and collaboration.

**Conclusions:**

A multi‐component Double‐Diamond design approach facilitated the development of primary care redesign priorities grounded in the reality of health systems and CBOs and offers a practical pathway for transitioning from co‐design to measurable QI efforts.

**Patient or Public Contribution:**

Community members and representatives from community‐based organizations (CBOs) were involved throughout the co‐design process as partners rather than as research participants. Community members and CBO representatives participated in the community engagement working group, contributed to fieldwork in high‐priority census tracts, and helped interpret findings from community health needs assessments and geospatial analyses to identify locally relevant priorities. Their lived experience informed the development of quality improvement (QI) priorities for clinic–community linked hypertension care. Stakeholder input focused on system‐level design considerations, rather than individual clinical encounters and will continue through a patient advocacy team composed of individuals living with hypertension from the target neighbourhoods to guide subsequent QI efforts. Coauthor Marquita Rockamore is a community member who leads a community health worker training programme at a local community college and serves as the chair of the steering committee for this work.

**Abbreviations**
ADI: area deprivation index
CBO: community‐based organization
CBPR: community‐based participatory research
CFIR: Consolidated Framework for Implementation Research
CHNA: community health needs assessment
CHW: community health worker
DD: double diamond
EHR: electronic health record
FQHC: federally qualified health centre
HRSN: health‐related social needs
mRFEI: modified retail food environment Index
PDSA: plan‐do‐study‐act
QI: quality improvement

## 1 Introduction

Hypertension remains one of the most prevalent and preventable causes of morbidity and mortality in the United States, and primary care systems struggle to consistently achieve effective and equitable hypertension control rates [1]. Neighbourhood‐level disadvantage is associated with a higher prevalence of hypertension and lower hypertension treatment rates [2]. Despite a growing evidence base for team‑based care and community‐engaged interventions to improve hypertension disparities, adoption in health systems continues to be challenging [3, 4].

Previous studies have often used community‐based participatory approaches to develop evidence‐based clinic‐community‐linked hypertension interventions [5, 6, 7, 8]. Recent studies highlight that redesigning hypertension care requires approaches that move beyond isolated interventions to multi‑level, contextually grounded redesign of workflows and system‑level processes. While human‐centred design has been applied to plan trial implementation strategies when developing interventions, co‐design extends human‐centred design principles into participatory care delivery redesign [9, 10, 11, 12, 13, 14, 15]. However, to our knowledge, few studies have applied structured co‐design frameworks to redesign quality improvement priorities in real‐world settings, a gap that limits the adoption and sustainability of redesign efforts at scale. Moreover, health systems serving socially disadvantaged neighbourhoods frequently confront parallel challenges such as medication affordability, transportation instability, mistrust, workforce constraints and inconsistent care transitions, yet improvement efforts remain isolated within individual health systems.

To address these gaps, we report the use of a co‑design approach guided by the Double Diamond framework to collaboratively identify primary care redesign priorities for hypertension care across four health systems serving an overlapping population with low socioeconomic status and a high prevalence of hypertension. We selected the Double Diamond framework because its structured divergence‐convergence phases are uniquely suited to early‐stage system redesign: the ‘Discover’ and ‘Define’ phases support broad exploration of lived experience and contextual barriers, while the ‘Develop’ and ‘Deliver’ phases guide teams toward synthesizing that complexity into actionable redesign priorities. Importantly, the four D's of the Double Diamond framework serve not only as sequential steps or phases but also as guiding principles that shape how teams engage with problems and stakeholders throughout the entire co‐design process. As principles, Discover orients teams toward curiosity and openness to unexpected findings; Define instils disciplined synthesis and a commitment to problem clarity before solution generation; Develop encourages creative, iterative exploration of potential redesign directions; and Deliver reinforces accountability to actionable, context‐grounded outcomes. This dual function, as both a process roadmap and a set of orienting principles, distinguishes the Double Diamond from more prescriptive QI frameworks and makes it particularly well suited to the kind of adaptive, multi‐stakeholder redesign required in complex, resource‐constrained health systems [16].

The Double Diamond framework differs from experience‑based co‑design by providing a clear structure to integrate multiple data sources, such as field observations, community health needs assessments (CHNAs), and geospatial analysis, in addition to stakeholder meetings across multiple health systems for collaborative priority‐setting in a shared, high‑need service region [17, 18]. Other co‐design approaches, such as the Patient Engagement Framework and Sanders & Stappers’ Co‐design Framework, differ fundamentally in their structure, scope and application to collaborative healthcare redesign, though all three support stakeholder involvement in improving care delivery. The Patient Engagement Framework describes engagement as an ongoing process across the care continuum, but does not specify transition to implementation [19, 20]. Sanders & Stappers’ framework emphasizes participatory methods but provides less explicit guidance for managing complexity across multiple health systems [21]. In contrast, the Double Diamond's structured divergence‐convergence method provided a clear and replicable approach uniquely suited to multi‐system collaborative redesign in a shared high‐need population—enabling teams to move from broad exploration to shared, actionable priorities in a way that neither alternative framework was designed to support.

The Double Diamond framework was necessary in this context because no existing implementation science or quality improvement framework provides the structured divergent‐convergent phases required to generate shared priorities across multiple health systems before problems are well‐defined—a precondition for meaningful co‐design in complex, multi‐organizational settings [22]. In selecting this framework, we also considered widely used implementation and quality improvement models. Frameworks such as Consolidated Framework for Implementation Research (CFIR), RE‐AIM (Reach‐Effectiveness‐Adoption‐Implementation‐Maintenance), and Normalization Process Theory are designed to guide the implementation and evaluation of interventions, rather than facilitate early‐stage exploration or collaborative generation of redesign priorities. While these frameworks are excellent for evaluating implementation, the Double Diamond is superior for the divergent exploration needed to define the ‘right’ problems in a complex, multi‐system environment. Similarly, traditional quality improvement approaches such as Lean, Six Sigma, and Plan‐Do‐Study‐Act cycles, are highly effective for optimizing well‑defined processes, but they are less suited for situations where the underlying problems are complex, context‑dependent problems requiring deep exploration before solution generation [23]. These QI frameworks help identify barriers and facilitators to implementation and evaluate implementation success, but they are not optimized for the divergent, exploratory work needed when problems are not yet well‐defined. These conditions require a co‑design approach that emphasizes divergent exploration before solution generation to identify the ‘right’ problems prior to testing solutions [24].

The need for structured codesign guidance is further underscored by recent work synthesizing coproduction, co‑creation and co‑design approaches (‘Three‑Cs’), which emphasizes the need for primary care‐specific frameworks and implementation guidance for co‐design methods [25]. Moreover, these approaches in primary care are often limited to single‑organization, small‑scale projects and focus only on patients as stakeholders, with very few studies reporting detailed methodological descriptions of co‑design processes [25, 26]. To our knowledge, no prior study has described multi‐system co‐design processes for a shared socioeconomically disadvantaged population with a high burden of uncontrolled hypertension, and prior applications of the Double Diamond framework typically focus on developing single interventions rather than system‐level priority alignment [16, 27]. We therefore report a formative co‐design phase of a multi‐stage QI initiative, guided by the Double Diamond framework, to collaboratively identify shared system‐level hypertension care redesign priorities across four health systems and partnering neighbourhood‐level community‐based organizations (CBOs) serving an overlapping, socially disadvantaged population with a high burden of hypertension. By applying a structured co‐design framework across multiple health systems and community partners, this study advances the codesign literature beyond single‐setting intervention development and offers a transparent account of how participatory design methods can inform cross‐system priority‐setting for chronic disease management in socially disadvantaged communities—a gap this study directly addresses. Additionally, by integrating geospatial, community, and stakeholder‐derived data to identify shared priorities, this work provides empirical evidence to support solution‐oriented, scalable system redesign, consistent with the National Institutes of Health's priorities for advancing sustainable approaches to health disparities. We describe our co‐design process as a novel way of developing quality improvement priorities that can be used by others planning regional efforts to improve health, and the specific shared priorities may be useful to others focused on cardiovascular health improvements in urban disadvantaged communities [12, 13, 14, 15, 28, 29]. The study has the following aims:

### 1.1 Aim 1

To describe collaborative generation of system‑level hypertension care redesign priorities across four health systems using a structured, Double Diamond–guided co‑design process.

### 1.2 Aim 2

To describe the feasibility and practical functioning of the Double Diamond framework as an umbrella structure for a multisystem co‐design process integrating multiple data sources (fieldwork, CHNAs, geospatial data and stakeholder insights) to facilitate the generation of shared hypertension redesign priorities for a high‐need, socioeconomically disadvantaged population.

## 2 Methods

### 2.1 Study Design

This study is a descriptive formative co‐design phase of a multisector hypertension care redesign initiative conducted across four urban health systems and community partners that provide care to our target population. The purpose of the formative codesign phase was to collaboratively identify system‑level hypertension care redesign priorities and was not intended to test clinical interventions. We used the Double Diamond design principles to structure stakeholder engagement and solution development, combined with rapid qualitative evaluation methods to synthesize insights in real time to inform the next steps of codesign processes. This project, including the co‐design activities, was approved as not human subjects research by the Institutional Review Board.

## 3 Setting and Participants

The co‑design working group included primary care clinicians, community health workers, community‐based organization members, population health leaders and system administrators from four urban health systems. We used the four key constructs when engaging multilevel stakeholders in the research process: (1) commitment of stakeholders to the process and the goals of the project; (2) capacity of stakeholders to participate in the process and engage in co‐design activities; (3) commitment of researchers to meaningfully engage stakeholders; and (4) trust among researchers and stakeholders [30]. This purposive sampling ensured representation of multiple perspectives on hypertension care delivery and lived experience. Participants were intentionally recruited to represent multiple sectors involved in hypertension prevention and management, including health systems, community organizations, public health agencies, academic institutions, payer organizations and population health collaboratives. See Table 1 for multisector representation in the co‐design process.

**Table 1:** Multisector representation in the co‐design process.

Sector and number of organizations	Participant roles	Number of participants
Health systems (*n* = 4)	Physicians; population health leaders; health system administrators; implementation scientists; quality improvement leaders; population health analytics leads; health system data specialists	16
Community organizations (*n* = 8)	Community programme directors, community health workers, community housing partners, food bank partners, YMCA representative, small business partners	11
Government and public health agencies (*n* = 1)	Public health programme administrators; chronic disease programme specialists	2
Academic partners (*n* = 2)	Health services researcher, data scientist, CHW programme director and faculty, trainee, informatics experts	6
Payer organizations	Medicaid payer representative	1
Regional quality improvement and population health collaboratives (*n* = 2)	Regional quality improvement evaluation specialists; population health collaborative leaders	4
Total individual participants: 40		
Total sectors represented: 6		

### 3.1 Data Collection and Data Sources

Data were collected through multiple sources during the co‐design process, including structured co‐design meetings, field observations and secondary data sources. See Table 2, inputs and data sources for co‐design process, which lists the data sources used to collect the information.

**Table 2:** Inputs and data sources for co‐design process.

Inputs	Co‐design data sources
Patient/community‐levels needs	Community engagement team co‐design meetings Community health needs assessments
Community‐level determinants	Field work in the community Community engagement co‐design meetings Geo‐mapping demographics of targe census tracts
Organizational needs	Data and evaluation team co‐design meetings
Organizational‐level determinants	Field work by the data and evaluation team to understand data‐level barriers and facilitators
External environment	Geo‐mapping of CBO and population demographics Payer and health systems participation in co‐design meetings

#### 3.1.1 Co‐Design Meetings

At the start of the project, two co‐design working groups were established: one for data and evaluation elements with participants primarily from health systems (*n* = 22), and the other for community engagement and health equity elements with participants primarily from the community (*n* = 18). Four health systems participated in the co‐design meetings, including a safety‐net university‐affiliated system, a federally qualified health centre (FQHC), a university‐affiliated health system and a large nonprofit integrated system. We conducted 14 structured co‐design meetings in total, including six data and evaluation working group meetings, five community engagement and health equity working group meetings, and three combined meetings of both working groups. Our first and last two meetings were combined meetings of both working groups.

##### 3.1.1.1 Double Diamond Co‐Design Approach and Activities

Skilled facilitators structured and guided each meeting to follow the four Double Diamond (DD) phases in sequence and ensured each meeting included time for reflection, discussion and consensus‐building across stakeholders, including in joint sessions where data‐evaluation and community engagement perspectives were integrated [31]. In the first two DD stages of discover and define, we worked with all stakeholders to understand the problem through the eyes of people (community, health systems, patients) who are affected by the issues to define the challenge and unmet needs. During the DD stages of develop and deliver, we facilitated the generation of solutions directly aligned with these unmet needs, prioritizing strategies for iterative testing through quality improvement cycles using the Plan–Do–Study–Act (PDSA) [32].

#### 3.1.2 Fieldwork by Working Groups

The community engagement working group completed walkthroughs of any one of our high‐priority census tracts to review structural and social determinants and the data and evaluation working group members met with one clinician and one community member to discuss actionable data that could improve cardiovascular health.

#### 3.1.3 Community Health Needs Assessments (CHNAs)

To inform the co‐design process and ensure that quality improvement priorities responded to locally identified needs, we conducted a review of existing CHNAs relevant to the census tracts prioritized for this initiative. We reviewed four existing needs assessments relevant to our high‐priority census tracts: two low‐income housing level needs assessments, our local county health needs assessment and a local county United Way descriptive summary of 211 calls for social needs. These reports incorporated secondary data analysis from regional community dashboard data, 211 service call data, community surveys, key informant interviews and focus groups.

Geospatial Analysis of priority and surrounding census tracts: We obtained sociodemographic and built‐environment characteristics for selected census tracts with high hypertension prevalence and their surrounding neighbouring census tracts. Variables included median income, area deprivation index (ADI), modified Retail Food Environment Index (mRFEI), non‐green space and other tract‐level characteristics. Data were collected at the census tract level using publicly available datasets.

### 3.2 Data Analysis

The primary outcome of this formative phase was the identification of shared hypertension care redesign priorities to be tested in QI initiatives across four health systems serving the priority population.

#### 3.2.1 Rapid Evaluation Methods

Given the iterative and time‐sensitive nature of the DD co‐design process, we employed a rapid qualitative analysis approach based on structured meeting summaries rather than full transcript coding. Rapid evaluation methods are increasingly used in health services research when findings are needed to inform ongoing implementation or quality improvement activities [33]. Rapid evaluation is described in the literature as frequently functioning as a precursor to longer‐term studies or as part of iterative evaluation cycles that inform ongoing system redesign [34]. In our study, the co‐design phase served as the formative stage of a broader quality improvement initiative, making rapid analytic approaches appropriate for synthesizing stakeholder insights in a timely manner to inform the next phase of the codesign process and QI initiative.

We used thematic analysis to examine co‐design meeting discussions and fieldwork notes in order to systematically identify recurring patterns in stakeholder perspectives on hypertension care redesign and health system improvement priorities [35, 36]. Thematic analysis was selected because it provides a rigorous yet flexible method for synthesizing data on a particular topic across diverse stakeholders and is well‐suited for applied implementation research focused on identifying actionable system‐level priorities rather than generating new theory, quantifying responses or exploring the lived experiences of individuals [37]. Our analytic approach aligns with methodological descriptions of rapid evaluation that increase analytic speed through streamlined data processing while maintaining analytic rigor [33, 34]. Specifically, structured meeting or topic summaries were used to synthesize key themes across co‐design sessions and integrate multiple sources of contextual data, including field observations, CHNAs and geospatial analyses. This approach enabled timely feedback to participating health systems and community partners while supporting cross‐session synthesis of emerging redesign priorities. Rapid analysis using structured summaries has been widely used in implementation and evaluation research when iterative feedback and decision support are required during ongoing programme development.

##### 3.2.1.1 Codesign Meeting Analysis

Co‐design meetings were recorded, and a structured meeting summary was created after every meeting by an expert in co‐design and project staff. Additionally, one author reviewed co‐design meeting summaries and recordings. To maintain rigor, structured summary templates for identifying recurrent patterns relevant to the project's objective were created with predefined domains (tracking and monitoring clinical measures, implementing team‐based care and linking community resources and clinical services). Each transcript was reviewed within these domains, and key quotations, observations, and action points were summarized in tabular matrices to enable comparison across meetings and working groups [38]. A thematic auditor reviewed all the co‐design meeting themes to ensure consistency with the data. We assessed themes guided by our main objective to identify priorities for co‐creating clinic‐community‐linked hypertension care services.

##### 3.2.1.2 Fieldwork Analysis

Fieldwork was analyzed using thematic analysis—two coders independently coded all transcripts, followed by collapsing the codes into potential themes and subthemes. A third qualitative expert reviewed all the coding and resolved any intercoder discrepancies. We used NVivo and Microsoft Excel to organize and compare codes. Intercoder reliability was ensured through double coding and consensus meetings to reconcile differences. Two thematic auditors reviewed all the fieldwork themes to ensure consistency with the data. We assessed themes guided by our main objective to identify priorities for co‐creating clinic‐community‐linked hypertension care services.

##### 3.2.1.3 Community Health Needs Assessments (CHNAs)

Key findings from the CHNA reports were reviewed and summarized descriptively by two coauthors to identify community priorities relevant to hypertension prevention, chronic disease management, and clinic–community linkages. The reports varied in methodology and data structure, so a narrative synthesis of priority needs and themes reported across the assessments was conducted. The synthesized findings were shared with community partners involved in generating these reports to validate interpretation and ensure alignment with community‐defined priorities for improving neighbourhood health.

##### 3.2.1.4 Geospatial Analysis of Priority and Surrounding Census Tracts

For the geospatial assessments, sociodemographic and built‐environment characteristics between our selected census tracts with high hypertension prevalence and surrounding neighbouring census tracts were described, and we derived counts for characteristics reported only as percentages using the total population as the denominator. Continuous ecological variables reported only as a single group‐level summary value (median income, ADI, mRFEI and non‐green space) were summarized descriptively because tract‐level variance was not available.

##### 3.2.1.5 Triangulation of Data to Identify QI Programme Opportunities

Information from all co‐design meetings, CHNA, field work and geomapping was summarized and triangulated by three authors to identify convergent themes and priority areas for hypertension care redesign. Triangulation also enhanced analytic credibility by examining the convergence and divergence of themes across independent data sources and stakeholder perspectives. Synthesized findings were reviewed in the final combined co‐design meetings to create a list of identified current state and potential opportunities to develop sustainable clinic‐community linked hypertension care services. The final co‐design meeting identified action steps to translate co‐design findings into QI programme opportunities.

## 4 Results

### 4.1 Geospatial Assessment of the Priority and Surrounding Census Tracts

Compared to the surrounding census tracts, our target neighbourhoods with a high prevalence of hypertension had a higher proportion of households living below 200% of the federal poverty level, without a vehicle, on food assistance and with a diagnosis of obesity and diabetes. See Table 3, demographic and socioeconomic characteristics of target and neighbouring census tracts.

**Table 3:** Demographic and socioeconomic characteristics of target and neighbouring census tracts.

Characteristic	Target census tracts *N* = 15 Total population 22,946		Neighbouring census tracts *N* = 127 Total population 259,568	
	*N*	%	*N*	%
Sex				
Female	12,382	54.0	140,648	54.2
Male	10,564	46.0	118,920	45.8
Race				
White	2279	9.9	56,780	21.9
Black	19,816	86.4	189,479	73.0
Other	851	3.7	13,309	5.1
Hispanic ethnicity	292	1.3	6013	2.3
Age, years				
18–40	2537	14.7	44,532	26.6
41–64	7866	45.6	81,968	49.0
≥ 65	6830	39.6	40,824	24.4
ADI mean		125		117
≤ High School Diploma	13,797	60.1	144,579	55.7
Households below 200% Poverty, %		64.9		54.7
Households with no vehicle, %		39.8		23.7
Receiving Food Assistance (SNAP)	5195	43.9	38,192	32.0
Modified Retail Food Environment Index (mRFEI)		4.4		9.1
Obesity, % of population		48.9		44.6
Diabetes, % of population		28.8		19.2
Hypertension, % of population		57.5		44.5

*Note:* Modified Retail Food Environment Index (mRFEI): A CDC measure ranging from 0 to 100, indicating the percentage of food retailers in a census tract that are considered healthy (e.g., supermarkets, produce stores) out of all food retailers. Lower scores reflect poorer access to healthy foods.

Abbreviation: SNAP = Supplemental Nutrition Assistance Programme.

### 4.2 Co‐Design Meeting Description and Themes

Community Health Needs Assessment Analysis: Across the four community needs assessments, consistent priorities emerged related to behavioural health, access to affordable healthcare and community conditions affecting health. Reports highlighted high mental health needs, barriers to affordable medical care and health literacy and structural determinants such as food insecurity, housing instability, neighbourhood safety and limited access to healthy food and health services. Social needs data indicated frequent requests for utility, housing and food assistance, while community surveys emphasized the importance of improved access to fresh food, healthcare services (including mental health care and pharmacies) and safer neighbourhood environments.

#### 4.2.1 Fieldwork Analysis

Fieldwork conducted by the data and evaluation working group identified challenges related to the actionability of patient‐generated health data, responsiveness to social needs questionnaires and measuring health behaviours at baseline and over time. Participants noted that existing health‐related social needs (HRSN) surveys capture information on diet, exercise and resource access but are often underutilized due to low response rates, particularly among patients who do not use patient portals, and because survey responses may not reflect dynamic changes in behaviours. Clinicians reported difficulty capturing reliable information about diet and physical activity in structured formats, suggesting that contextual measures such as proximity to food sources or exercise spaces may be easier to operationalize. Community walkthroughs identified environmental factors that could influence cardiovascular health, including variability in housing conditions, limited healthy food options relative to convenience stores and fast‐food outlets, transportation infrastructure that may affect access to services, conditions of sidewalks and uneven distribution of healthcare facilities, pharmacies and recreational spaces. Together, these findings informed the co‐design process by highlighting the importance of integrating community context, improving methods for capturing actionable social and behavioural data and identifying priorities that address both clinical care and structural barriers to healthy behaviours.

#### 4.2.2 Codesign Meeting Analysis

Using an iterative rapid qualitative synthesis approach, recurring themes across co‐design meetings and stakeholder groups were consolidated by the Double Diamond phase to capture the progression from problem identification to actionable implementation strategies (Table 4).

**Table 4:** Overarching co‐design meeting discussions by codesign phase.

Double diamond phase	Data and evaluation working group—key findings	Community engagement and health equity working group—key findings
Discover: Unstructured findings	Identified gaps between patient‐generated health data (e.g., home monitoring, lifestyle behaviours) and clinical EHR data, limiting the ability of care teams to monitor hypertension and related behavioural risk factors. Participants emphasized the need to integrate clinical, social needs and geospatial data into unified dashboards to support population health management and quality improvement.	Identified existing community assets and partnerships and emphasized the importance of understanding community conditions influencing health. Participants highlighted food access, transportation and neighbourhood safety as key determinants affecting hypertension prevention and chronic disease management.
Define: Opportunities	Prioritized opportunities to align clinical and social risk screening with actionable care processes. Participants emphasized reducing redundant screening, linking data collection to medication management and care coordination, and leveraging external data sources to support integrated care delivery.	Defined cross‐cutting community priorities based on CHNA findings, including food insecurity, access to healthcare services and neighbourhood safety. Participants emphasized strengthening connections between clinical care and community resources and expanding team‐based care models involving community health workers.
Develop: Many ideas	Generated strategies to operationalize referral systems for social needs, standardize data collection across health systems and develop interoperable platforms for sharing screening and referral information. Participants emphasized the importance of actionable data dashboards and geospatial targeting to guide outreach and quality improvement.	Developed community‐centred implementation strategies, including expanding nutrition education and food access initiatives, strengthening referral pathways to community resources and engaging community health workers embedded within communities. Participants emphasized reducing administrative burden and ensuring technology platforms are accessible to community organizations.

The first co‐design combined team meeting introduced the project goals, team members and explored the current overlapping activities in the targeted neighbourhoods. Participants made recommendations on relevant recently completed CHNAs, and mapping census tracts to identify and visualize food pantry, healthcare and pharmacy locations. Additionally, the co‐design team members recommended exploring partnerships with Foodbank partners, school health programmes, the Urban Barber Association, congregations and faith‐based organizations, as well as CBOs focused on housing, legal aid, literacy and aging populations.

##### 4.2.2.1 Discover Stage

###### 4.2.2.1.1 Data and Evaluation Working Group

During the first two meetings of the discover stage of DD, the data evaluation working group identified gaps between patient‐generated health data (lifestyle behaviours, self‐monitoring) and clinical EHR data, which limit team‐based care teams from monitoring progress on blood pressure control and behavioural metrics across care settings. The second meeting focused on unstructured findings, including the need to standardize and consolidate all existing data into a single visualization or dashboard, addressing delays in data extraction to facilitate timely continuous quality improvement, and the requirement to integrate additional data as it evolves over time. Codesign participants identified the need for streamlining geomapping and the potential for de‐duplication across larger EHR‐based platforms.

*Example quotes from stakeholders:*

1. *‘. . the use of home monitoring devices and looking at those trends, not only when the patient is in with their provider, but also how they are using these tools at home. That dovetails into another question: when the patient is in for a visit, how can we use the clinical team to assess how well that patient understands or finds it useful to use those tools, whether it's electronic tools that capture their steps or non‐electronic ones like food logs?’*
2. *‘we deploy. . survey. . mainly via my chart, and individuals who live in areas that are more disadvantaged might have less access to my chart…’*
3. *‘. . we actually really need to combine blood pressure cholesterol data, clinical EHR data and social needs data like, what are the social needs and those census tracks. That's the data we're going to be visualizing together across this to really drive improvements.’*

###### 4.2.2.1.2 Community Engagement Working Group

In the first meeting, working group members felt it would be logical to align project goals with current ongoing active community partnerships and projects in the target census tracts, such as community health worker (CHW) led interventions, city recreation centre improvements, ward‐level asset mapping, bike programmes and BP monitoring programmes in the community. In the second meeting, the need for layered data on hypertension, food access, transportation and social risk was suggested. Access to healthy food was frequently mentioned as an unmet need in the target communities.

*Example quotes from stakeholders:*

1. *‘none of the foods were particularly healthy. .(in the neighbourhood store) There is frozen food, prepackaged food, canned and bottled food and condiments, desserts, and hot food.’*
2. *‘community health work is not always about food, insecurities or food deserts. It's also about what you want your community to look like. If you want it to be safe…if there's lights out… that's important. Community condition is very important.’*
3. *‘Update census tract map to include parks and community gardens Add Bike CBO to list of partners for potential outreach. The City has capital improvement projects underway at many of its recreation centers. We should include those activities’*

##### 4.2.2.2 Define Stage

*Data and Evaluation working group:* After exploring needs in the first two meetings, the working group's third meeting defined shared goals of avoiding screening that is not actionable, aligning social needs and clinical data collection with medication management, and leveraging existing external data sources to support integrated care coordination.

*Example quotes from stakeholders:*

1. *‘people are being over‐screened (for social risks) and I mean, they're oftentimes being asked the same questions by multiple entities. There's a lack of sort of understanding of why they're being asked certain questions and a desire of like of kind, of being dropped off on some of these referrals of sort of like. Well, someone will follow up with you, and nothing ever happens’*
2. *‘Yeah, I guess the question deals with getting a lot of data on everyone, with the exception of the providers… Providers use some medications, and a lot of this (improving cardiovascular risks), particularly for things like hypertension, depends on the number of meds and the intensification of treatment.’*

###### 4.2.2.2.1 Community Engagement Working Group

In the third meeting, the community engagement group identified overlapping goals of addressing food insecurity, access to health care and safety in the community based on the review of CHNAs. The workgroup defined the shared goals of collecting data in community settings, linking clinical care with community services, and team‐based care.

*Example quotes from stakeholders:*

1. *‘Some of the work we're doing is looking at some of the food that's available with the food pantries and things like that…. . because I know some of it doesn't look appealing a lot of times and some of the people haven't seen so providing engaging nutritionist to the clinics and dietitians, and what this food is kind of like some educational pieces around that, too, and what you some different things that you can do with it.’*
2. *‘I was thinking, why don't we go to a lot of the people in these communities …with like a transportation bus. I would love to have like maybe a health worker on the bus with me doing blood pressure. You know, testing. And you know, maybe someone from the food bank when we have food on the bus. We can travel to the barbershops all around the city,…’*
3. *‘It is usually about cost and ability to get (prescription) in an economy manner. So when I think about the pharmacies around that area. They are truly overwhelmed. If you haven't visited them, there's no, there's not a lot of education. It's do you have any questions? Nope, okay. And it's onto the next… having community health worker's ‐ People who are there can answer, you know, can help them where there's a cost issue or transportation issue, and not promoting any one chain’*

##### 4.2.2.3 Develop Stage

###### 4.2.2.3.1 Data and Evaluation Working Group

During the fourth meeting, ideas were generated focused on realistic referral systems for social needs that are based on service capacity, capturing data from referral platforms and internal referrals, activating technical functionality to share social needs screening data across health systems, and balancing resources for in‐person screening and coordination versus combining geomapping and healthcare data for electronic risk stratification. In the fifth meeting, most health systems mentioned ongoing work in EHR‐integrated screening and referrals for social needs to reduce provider burden, but noted the need for tailoring the process based on patients’ risk levels and the importance of patient consent and warm handoffs to improve patient engagement in the referrals. The sixth meeting focused on defining reporting strategies as a multisystem group for enhanced interoperability, staff‐motivated screening workflows for clinical and social needs data, and building centralized, actionable geospatial dashboards to guide outreach and resource deployment.

*Example quotes from stakeholders:*

1. *‘we would need to make sure that all the systems are sort of synchronized, or at least we standardize the responses. And you know, definitions. Cause they may vary. You know the way that questions are coded, and the points and such. And the way that the need is determined may be different across different sites. We should just make sure that we're measuring the same thing in the same way.’*
2. *‘things that we've seen is when we get a warm referral versus a cold referral. So cold referral, we're likely to have a conversion rate of somewhere between 10 and 15(%). But a warm referral. It's more in the forties(%…Cause you imagine that you're going about your normal day and someone's calling you that you don't know. And we all know that there's a lot of spam out there…’*
3. *‘The Sky with Stars program. . a staff led initiative. . came from our medical assistants and our nurses. . it's working to just inspire the individuals who see their care …. they have a big billboard, our big board that the patients can walk through as they as they aspire, their star goes up as they reach their goal…….’*

###### 4.2.2.3.2 Community Engagement Working Group

During the fourth meeting, the CE WG brainstormed ideas for aligning ongoing health system‐level activities to meet needs identified through CHNA reviews. Leveraging facilitators of referral platforms for screening and referral to community resources and hubs was suggested. Access to nutrition education and healthy foods (e.g., food banks), and engaging CHWs from the community (not just employed by health systems) were identified as important strategies. Fifth meeting discussions focused on reducing the burden associated with fragmented systems, screening using QR codes at community or mobile unit sites, and user‐friendly platforms that can be affordable for small community organizations.

1. *‘it really, for our team comes down to double data entry, receiving and sending referrals via some of these platforms means we're entering the data into our system and into another system. And that involves, you know, more time or lost data entry, because folks just don't remember in into 2 places. And so something to support that the integration between the 2 systems could be really helpful for an organization like ours.’*
2. *‘People wouldn't have to, you know, find ways to go all the way outside their community for that assistance, because the mobile unit can come to them, and they can do most of what's you know what's needed on that list, anyway…that would be a great way to kind of gather and consolidate that data and make it more accessible to the community at the same time and improve that trust.’*

##### 4.2.2.4 Deliver Stage

The final two combined meetings focused on refining solutions and outlining follow‐up quality improvement (QI) activities to operationalize the co‐designed strategies. Meetings identified a need to conduct asset mapping to identify existing community assets and align CBOs with specific SDoH domains to prioritize initial outreach and engagement in the collaborative.

The last combined meeting focused on summarizing all the co‐design activities and transitioning to QI priorities. Participants converged on feasible action steps and prioritized opportunities for QI initiatives such as cross‐system team‐based care and collaboration; improving data usability and standardizing data across health systems; improving data interoperability and the real‐time availability of CBO resources; and incorporating the lived experiences of a patient team in QI strategies. Key themes from these meetings are summarized in Table 5.

**Table 5:** Deliver stage—integrated co‐design themes from final combined meetings.

Delivery stage theme	Key findings from combined co‐design meetings
Strengthening clinic–community referral infrastructure	Participants identified variability in social needs screening practices and referral pathways across health systems and community organizations. Stakeholders emphasized improving interoperability of referral platforms, aligning referral workflows with community organization capacity and supporting community‐based organizations’ access to referral systems to strengthen clinic–community linkages.
Integrating community health workers into care models	Community health workers were widely recognized as essential for bridging clinical and community care. Proposed roles included supporting social needs screening, facilitating referrals, assisting with hypertension self‐management and providing navigation support for patients across healthcare and community settings.
Aligning community engagement with health system initiatives	Participants recommended asset mapping to identify community‐based organizations and align them with specific social determinant domains. Stakeholders emphasized leveraging existing community programmes and strengthening partnerships with food access initiatives, community health programmes and neighbourhood organizations.
Developing implementation‐ready QI strategies	The final co‐design discussions focused on translating identified priorities into actionable quality improvement strategies, including improving team‐based hypertension care, optimizing social needs screening workflows, strengthening referral loops between clinics and community organizations and developing geospatial dashboards to guide outreach and population health management.
Evaluation and sustainability planning	Stakeholders discussed evaluation strategies including grant‐funded evaluation and health system performance measurement. Proposed measures included clinical process indicators and metrics assessing the effectiveness of clinic–community referral pathways.

## 5 Discussion

By documenting a Double Diamond‑guided co‑design process that spans four health systems serving the same socioeconomically disadvantaged population with high hypertension prevalence, our study advances this literature in several ways. First, we offer a transparent description of how codesign principles were operationalized across organizational boundaries, including stakeholder selection, data integration (fieldwork, CHNAs, geospatial analyses), and structured prioritization. Second, we demonstrate how co‑design can move beyond the development of single interventions to identify system‑level redesign priorities that are shared across institutions and grounded in local context. Third, our descriptive account lays the groundwork for the kind of implementation guidance called for in recent reviews by making explicit the steps, structures, and design decisions involved in conducting a multi‑system co‑design process in a resource‑constrained, high‑need region [25]. Although we do not assess long‑term sustainability in this phase, our approach provides a replicable model that future work can build on to evaluate implementation and impact.

Prior studies have used co‑design and human‑centred design methods to develop specific interventions, but they seldom address multisystem challenges or the structural barriers that impede sustained improvements in hypertension care. Our study demonstrates the added value of applying a Double Diamond approach to identify system‑level redesign priorities rather than isolated interventions. By integrating insights from co‑design meetings with four local health systems and community members, fieldwork observations, CHNAs and geospatial analyses, we generated a more comprehensive understanding of the factors shaping hypertension management across clinics and communities. This multi‑source process allowed us to triangulate findings and avoid overreliance on any single perspective, ensuring that the resulting priorities reflect the dynamic realities of primary care work systems and the needs of the populations they serve.

Our co‐design process generated four cross‐cutting priorities for primary care redesign: improving data usability and interoperability, incorporating lived experience into redesign decisions, enhancing real‐time visibility and coordination of CBO resources and strengthening cross‐system team‐based care and health system‐community collaborations. These priorities are similar to key constructs used in implementation and quality improvement frameworks. For example, improving shared referral platforms, geospatial dashboards and data interoperability are inner and outer setting factors influencing the implementation of clinic‐community linked services, aligned with the CFIR, while embedding CHWs and partnering with CBOs to support hypertension care aligns with the recommended clinic‐community linked team‐based care strategies recommended by national hypertension initiatives [39, 40, 41, 42, 43].

Our findings have important implications for clinical practice and health system operations. Our co‐design process generated practical redesign priorities that were not characteristic of a single health system or model but instead reflected shared needs and priorities across a local network of healthcare systems and community partners working in settings with a high burden of uncontrolled hypertension. While many health systems reported progress in embedding social needs screening into EHRs, our participants identified opportunities for shared referral tracking and centralized dashboards across local health systems to guide outreach and resource deployment. These findings suggest that sustainable clinic‐community integration will require a coordinated multisystem investment in tools that are not only technically interoperable but also co‐designed with end users to be functionally meaningful in both health system and CBO contexts. By providing a structured method for integrating frontline clinical perspectives with community‐level data, the Double Diamond‐guided co‐design approach can serve as an early‐stage step in larger quality improvement and implementation efforts aimed at improving hypertension control in socioeconomically disadvantaged communities.

### 5.1 Limitations

Our study has several limitations. Our findings are specific to one urban Midwestern county and may not generalize to other demographic or organizational structures. Nevertheless, our participating health systems represented diverse health systems (a safety net system, an FQHC network, a university health system and a nonprofit, large integrated health system). Second, while the Double Diamond framework provided structure for inclusive ideation, the translation of design ideas into measurable impact remains in the early stages. Implementation and outcome data will be critical in evaluating the success and scalability of proposed QI interventions. We did not collect feedback surveys from co‐design participants to reduce the burden of participation, as in addition to attending co‐design meetings, participants were also engaged in reviewing additional information or reporting from communities and health systems. Lastly, although we triangulated fieldwork, geospatial assessments, needs assessments and stakeholder input, the data were primarily qualitative. Future phases will incorporate quantitative measures to assess the adoption and impact of clinic‐community linkages.

### 5.2 Next Steps

The transition to QI will be guided by a key driver diagram and the Plan‐Do‐Study‐Act (PDSA) framework. Health system teams will strategically engage with community partners, as required for QI initiatives. We established a patient advocacy team composed of individuals living with hypertension from our target neighbourhoods to ensure QI priorities remain grounded in patient realities and priorities. The review of CHNAs was aligned with ongoing health system implementation strategies, helping to define shared goals and identify feasible entry points for QI efforts. Quality improvement will focus on effective communication between health systems and CBOs, treatment (of medical and social needs) for at‐risk individuals, and engagement with community partners in compatible initiatives (e.g., addressing food insecurity and nutrition education in food pantries, increasing hypertension screening, awareness and primary care linkages via barbershop initiatives).

## 6 Conclusion

Our Double‐Diamond co‐design strategy brought together four local health systems, CBOs and other key stakeholders to collaboratively identify priorities for redesigning hypertension care for high‐risk communities. By applying a human‐centred, systems‐oriented approach, we uncovered critical, actionable barriers to clinic‐community linked hypertension care and co‐developed opportunities for care redesign. Next, our data‐driven quality improvement efforts will be guided by our co‐design findings and grounded in the lived experiences of our patient populations. Through structured evaluation and continuous stakeholder engagement, we aim for sustainable primary care redesign to address the persistent local gaps in hypertension care delivery.

## Author Contributions

**Sonal J. Patil:** conceptualization, writing – original draft, methodology, formal analysis, writing – review and editing, data curation, investigation, supervision. **Aleece Caron:** funding acquisition, conceptualization, methodology, writing – original draft, writing – review and editing, supervision, investigation, resources, formal analysis, project administration. **Mary Joan Roach:** formal analysis, supervision, writing – original draft, writing – review and editing. **Douglas Einstadter:** formal analysis, methodology, writing – original draft, writing – review and editing, data curation, investigation, supervision. **Shelia Malone:** data curation, writing – review and editing. **Quisha Marbury:** formal analysis, writing – original draft, writing – review and editing. **Morgan Whaley:** formal analysis, writing – original draft, writing – review and editing. **Catherine Sullivan:** project administration, writing – original draft, writing – review and editing, formal analysis. **Perry J. Kimmel:** formal analysis, project administration, writing – original draft, writing – review and editing. **Marquita Rockamore:** supervision, writing – original draft, writing – review and editing, project administration. **Christopher Mundorf:** writing – original draft, writing – review and editing, supervision, data curation. **Kipum Lee:** methodology, supervision, investigation, conceptualization, writing – original draft, writing – review and editing. **Shari D. Bolen:** conceptualization, investigation, funding acquisition, writing – original draft, writing – review and editing, validation, supervision, resources, methodology, project administration, formal analysis.

## Ethics Statement

All procedures were in accordance with ethical standards. The MetroHealth IRB determined this project to be Not Human Subjects Research but a Quality Improvement project.

## Consent

The authors have nothing to report.

## Conflicts of Interest

The authors declare no conflicts of interest.

## Data Availability

Data are available on request with approvals.
